# Erratum for Mor et al., “Identification of a New Class of Antifungals Targeting the Synthesis of Fungal Sphingolipids”

**DOI:** 10.1128/mBio.00188-18

**Published:** 2018-03-13

**Authors:** Visesato Mor, Antonella Rella, Amir M. Farnoud, Ashutosh Singh, Mansa Munshi, Arielle Bryan, Shamoon Naseem, James B. Konopka, Iwao Ojima, Erika Bullesbach, Alan Ashbaugh, Michael J. Linke, Melanie Cushion, Margaret Collins, Hari Krishna Ananthula, Larry Sallans, Pankaj B. Desai, Nathan P. Wiederhold, Annette W. Fothergill, William R. Kirkpatrick, Thomas Patterson, Lai Hong Wong, Sunita Sinha, Guri Giaever, Corey Nislow, Patrick Flaherty, Xuewen Pan, Gabriele Vargas Cesar, Patricia de Melo Tavares, Susana Frases, Kildare Miranda, Marcio L. Rodrigues, Chiara Luberto, Leonardo Nimrichter, Maurizio Del Poeta

**Affiliations:** a Department of Molecular Genetics and Microbiology, Stony Brook University, Stony Brook, New York, USA; b Department of Chemistry and Institute of Chemical Biology and Drug Discovery, Stony Brook University, Stony Brook, New York, USA; c Department of Biochemistry and Molecular Biology, Medical University of South Carolina, Charleston, South Carolina, USA; d Department of Veterans Affairs Medical Center, Cincinnati, Ohio, USA; e University of Cincinnati College of Medicine, Cincinnati, Ohio, USA; f Department of Pharmaceutical Sciences, University of Cincinnati, Cincinnati, Ohio, USA; g Department of Pathology, Fungus Testing Laboratory, University of Texas Health Science Center at San Antonio, San Antonio, Texas, USA; h Division of Infectious Diseases, University of Texas Health Science Center at San Antonio, San Antonio, Texas, USA; i Department of Pharmaceutical Sciences, University of British Columbia, Vancouver, British Colombia, Canada; j Department of Biomedical Engineering, Worcester Polytechnic Institute, Worcester, Massachusetts, USA; k Department of Biochemistry and Molecular Biology, Baylor College of Medicine, Houston, Texas, USA; l Instituto de Microbiologia Professor Paulo de Góes, Universidade Federal do Rio de Janeiro, Rio de Janeiro, Brazil; m Laboratório de Ultraestrutura Celular Hertha Meyer, Instituto de Biofísica Carlos Chagas Filho, Universidade Federal do Rio de Janeiro, Rio de Janeiro, Brazil; n Diretoria de Metrologia Aplicada a Ciências da Vida, Instituto Nacional de Metrologia, Qualidade e Tecnologia (INMETRO), Xerém, Rio de Janeiro, Brazil; o Centro de Desenvolvimento Tecnológicoem Saúde (CDTS), Fundação Oswaldo Cruz (Fiocruz), Rio de Janeiro, Brazil; p Department of Physiology and Biophysics, Stony Brook University, Stony Brook, New York, USA; q Department of Chemistry, University of Cincinnati, Cincinnati, Ohio, USA

## ERRATUM

Volume 6, no. 3, e00647-15, 2015, https://doi.org/10.1128/mBio.00647-15. The compound that was used in our article was *N*′-(3-bromo-6-hydroxybenzylidene)-2-methylbenzohydrazide (BHBM) and not *N*′-(3-bromo-4-hydroxybenzylidene)-2-methylbenzohydrazide. All experiments described in the paper were done using BHBM [*N*′-(3-bromo-6-hydroxybenzylidene)-2-methylbenzohydrazide], and the structure noted in Fig. 1B of our original article should be as indicated in Fig. 1 here.

**FIG 1:**
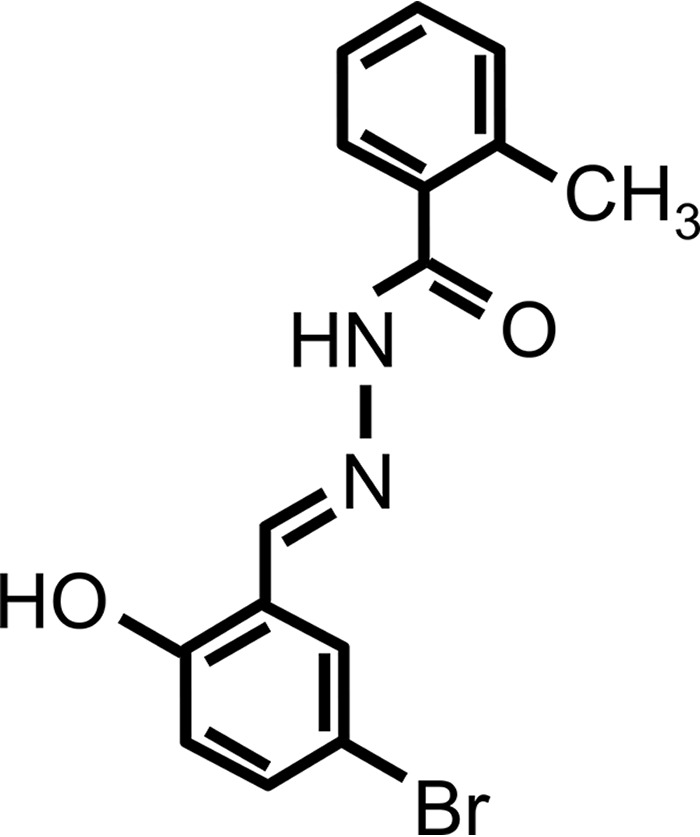
Corrected structure of BHBM to replace the structure in Fig. 1B of our original article.

The structure of BHBM used in the *mBio* studies was confirmed by proton nuclear magnetic resonance (NMR), which indicated the following chemical shifts: ^1^H NMR (500 MHz, dimethyl sulfoxide-d6) δ 2.38 (s, 3H), 6.90 (d, 1H, *J* = 8.8 Hz), 7.28 to 7.32 (m, 3H), 7.37 to 7.43 (m, 2H), 7.47 (d, 1H, *J* = 7.5 Hz), 7.78 (s, 1H), 8.47 (s, 1H), 11.19 (s, 1H), 12.05 (s, 1H).

All findings illustrated in the paper are correct.

